# Enhancing Medical Student Resilience and Wellness: Assessing Acceptability of a Supportive Student-Alumni Network

**DOI:** 10.1007/s40596-025-02164-0

**Published:** 2025-06-26

**Authors:** Delaney Schrenk, Aydin Kaghazchi

**Affiliations:** University of Cincinnati College of Medicine, Cincinnati, OH USA

**Keywords:** Medical student, Wellness, Burnout, Attrition, Resilience

## Abstract

**Objective:**

This pilot study assessed the acceptability of an online network where medical students could seek advice from alumni regarding social and academic challenges. Objectives included assembling a critical mass of students and alumni who would be interested and determining if alumni’s past experiences overlap with current student challenges and what communication modalities both parties would use.

**Methods:**

A survey was sent to first-, second-, and third-year medical students at the University of Cincinnati to gauge interest and to see which experiences they would want support for. It was advertised through emails, newsletters, posters, and social media. A similar survey was sent to alumni who graduated in the past 5 years. Responses were collected over 3 months.

**Results:**

Responses from 78 of 535 (15%) students and 36 alumni were collected, with 71% (55/78) of students and 91% (33/36) of alumni being interested. The most common topics for both groups were burnout, depression, anxiety, loneliness, and ending a relationship. There was at least one alumnus for each experience. Of 31 alumni and 57 students who answered how to meet, 61% of alumni preferred phone for first contact, and 85% of students preferred virtual meetings.

**Conclusions:**

The results suggest that creating a student-alumni network was acceptable due to similar demographics and overlapping experiences. The data indicated that students could contact alumni by phone to discuss how to meet given the diversity of student preferences. This resource could be explored at other medical schools as a novel means of support.

Medical students face challenges on both personal and professional levels. As a result, medical students have a higher prevalence of depression, anxiety, and psychological distress than the average population [1]. Many studies focus on the academic factors of that distress. However, national data regarding medical student attrition show that 1.8% of students withdraw for non-academic reasons, while 1.2% of students withdraw for academic reasons [2].

One way to cope with both types of stressors is having adequate social support [3, 4]. This support is especially important because lack of social support contributes to burnout, which is associated with serious thoughts of taking a leave of absence [1, 5–7]. In medical school, social support can often take the form of mentorship.

Mentorship is highly desired by medical students to both cope with stressors and debrief after they occur [4, 8]. Near-peer mentors have additional value because educators often have difficulty discerning which students are starting to struggle and because students tend to seek help from peers, rather than educators [9, 10]. The literature regarding near-peer mentorship currently focuses on academic success, as opposed to interventions that address social stressors. Even fewer studies define near-peers, as alumni and alumni mentorship have only been studied in cohorts of nursing students. In these studies, the goal was for alumni to help nursing students transition to the workforce via monthly casual meetings. Nursing students gained personal and professional benefits while mentors felt satisfaction in helping them in their journey [11, 12].

This study was initiated by medical students to address the paucity of research surrounding alumni mentorship. Specifically, the authors propose a student-alumni network where medical students have access to recent alumni to privately discuss shared social challenges during their medical education. It is also an opportunity to confidentially share struggles with mental health as stressors arise during school. This intervention would hopefully fill a gap in support for students who are not ready to seek formal counseling, especially since most leaves of absence occur during the first year [6]. If acceptable, this may be an initiative to explore at this institution and potentially others.

## Methods

Following institutional review board approval, two Qualtrics surveys were developed (Qualtrics, Provo, UT). Consent information sheets were provided at the beginning of both surveys.

The student survey was available to current first-, second-, and third-year medical students at the University of Cincinnati. Fourth-year students were excluded because the network would not be available until after they graduated. It was sent to students via email and accessible via a QR code on printed flyers posted in campus common areas. It was also listed on the school newsletter and the Instagram page of the student wellness committee. One month after initial emails, it was advertised in person to first- and second-year students at a mandatory lecture. A reminder email followed the in-person advertisements.

The survey asked students to identify demographics (e.g., gender, ethnicity), indicate interest, and share factors that influenced their interest in this resource. It also asked students to select challenges for which they would want support. The selectable challenges included failing a course/board exam, considering a leave of absence, financial hardship, divorce/ending a relationship, becoming a parent, grief, burnout, loneliness, depression/anxiety, chronic illness, disability, neurodivergence, other mental health concern, and “other” with a free-text space for students to add any challenges that were not listed. A final question asked students to select all modalities they would be willing to use to contact alumni, including a phone call or video call or in-person. Responses were collected over 3 months.

The alumni survey was sent to both the general alumni emailing list and the emailing list for Gold Humanism Honor Society members who graduated in the past 5 years. It was sent to alumni email addresses twice through the Alumni Engagement Office per institutional policy. The original and reminder emails were separated by 1 month.

This survey asked alumni to identify demographics (e.g., gender, ethnicity), indicate interest, and share factors that influenced their interest in this resource. Like the student survey, it asked alumni to select any of the challenges listed above and offered a free-text space for alumni to add any unlisted challenges. It also asked which method of communication was most preferred (phone call, video call, or in-person). There were two questions unique to the alumni survey. Only the alumni survey asked what supports they utilized when navigating challenges in medical school, and it allowed participants an option to provide their names and contact information (e.g., phone, email address) with the anticipation of generating a student-alumni network. Responses were collected over 3 months.

For both surveys, the selectable challenges were chosen from the risk factors for attrition from medical programs [13–15].

## Results

Responses were collected from 78 students (response rate of 15%) and 36 alumni. Responses were excluded if participants did not answer the question regarding their level of interest in a student-alumni network. For those who did answer this question regarding interest, 71% (55/78) of students selected yes, and 24% (19/78) of students selected “maybe.” Students who chose “maybe” would be more interested if they found personal time to meet with alumni, had guidance in navigating the network, developed unique hardships later in training, or could talk to non-traditional students. Only 5% (4/78) were not interested.

Of the 36 alumni, 91% (33/36) were willing to talk to students, and 6% (2/36) selected “maybe.” Only 3% (1/36) of alumni were not interested. Table 1 displays the demographics of the students and alumni who responded to the survey.

**Table 1:** Demographics of student and alumni survey respondents

Item	Students (*N* = 78)	Alumni (*N* = 36)
Gender	*n* (%)	*n* (%)
Female	47 (62%)	21 (60%)
Male	29 (35%)	13 (37%)
Non-binary	1 (1%)	2 (3%)
Undisclosed	1 (1%)	0 (0%)
Race/ethnicity	*n* (%)	*n* (%)
White/Caucasian	49 (64%)	26 (71%)
Black/African American	6 (8%)	4 (11%)
Hispanic/Latinx	10 (14%)	1 (3%)
East Asian	6 (7%)	2 (6%)
South Asian	12 (15%)	6 (17%)
Native American	0 (0%)	2 (6%)

**Fig. 1:**
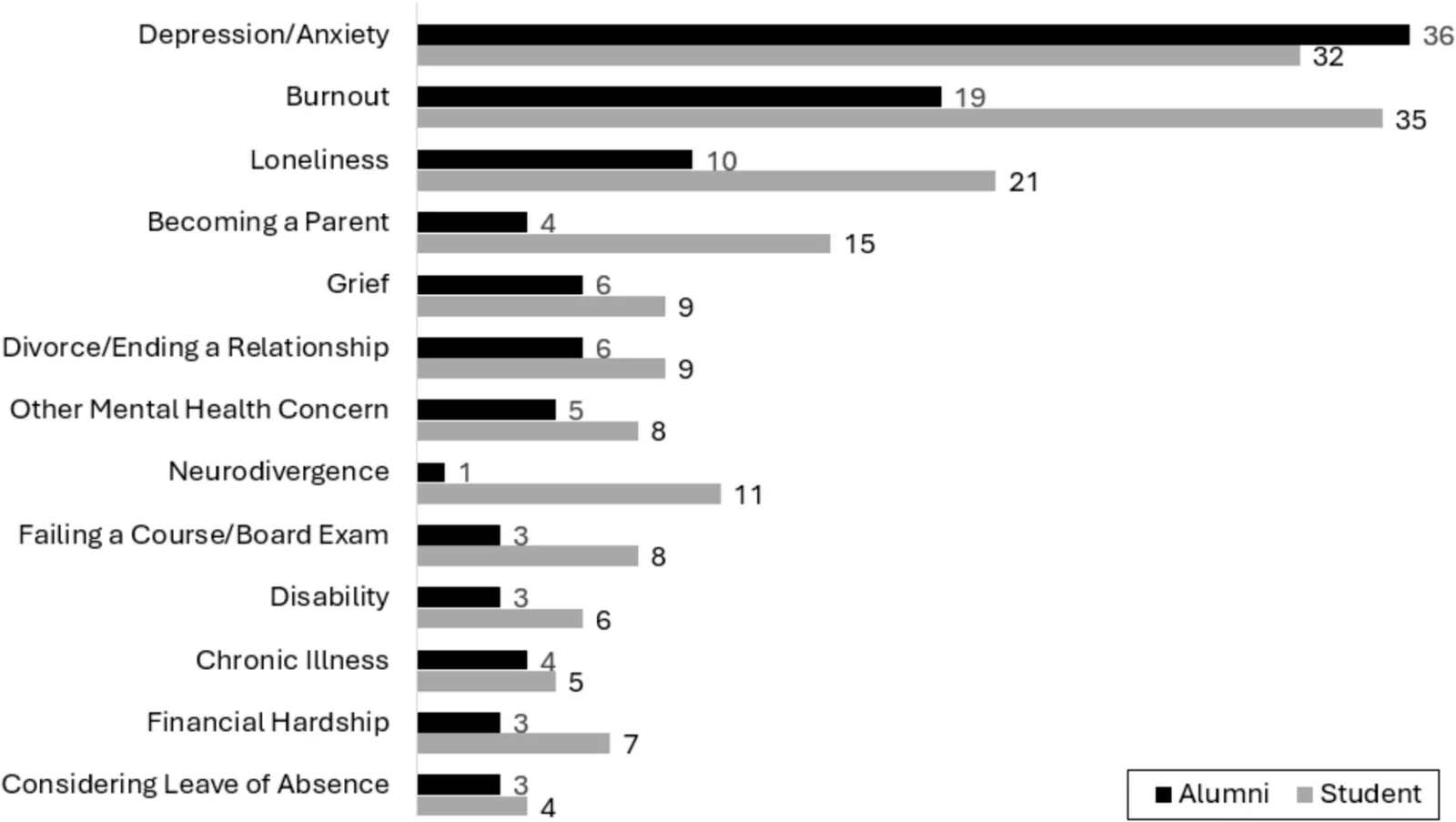
Medical school challenges experienced by students (*N* = 60) and alumni (*N* = 35). Respondents were allowed to select multiple applicable experiences

In both surveys, participants selected all challenges that applied to them. A total of 60 students and 35 alumni reported experiences that they would have wanted support for. Students also used the free-text space to submit additional experiences that they would want support for. These experiences included first-generation challenges, non-traditional student challenges, long-distance relationships, military scholarship commitments, being a primary caregiver, and loss of a patient/running codes. Unlisted experiences added by alumni included imposter syndrome, being a minority in medicine, and friend or family difficulties. Figure 1 shows the complete findings from both surveys.

In the alumni survey, 80% (28/36) of the respondents selected yes to “As a medical student, did you experience anxiety regarding reaching out for help?” and 93% (33/36) selected yes to “Does that prior anxiety motivate you to participate in this Network?”.

A total of 57 students and 31 alumni answered how they would connect for a meeting. Virtual modalities were preferred by 84% (48/57) of students and 23% (8/31) of alumni. In-person meetings were preferred by 72% (41/57) of students and 16% (5/31) of alumni. Phone calls were preferred by 53% (30/57) of students and 61% (19/31) of alumni.

## Discussion

Offering a formal database of alumni with whom to connect regarding personal challenges is a novel way of supporting medical students. As a form of near-peer support, it may decrease isolation and improve resilience [8]. Established physicians disclosing their own struggles may also encourage medical students to seek help [16]. Therefore, this resource is especially useful for those who may not be ready for formal care or who temporarily withdraw from medical programs where there is less institutional facetime.

The results from this study suggest that this is a desired and acceptable resource based on our three main objectives. First, most respondents of both parties indicated interest. Second, the challenges and demographics of students and alumni overlapped well. Finally, communication preferences suggest that meetings can be initially arranged via phone calls per alumni preference and take place virtually given student preference.

With these three conclusions in mind, it is important to discuss some caveats regarding the sample size. Although the student response rate was 15%, there is a national estimate of 11% of medical students who consider withdrawing annually [7]. Our response rate therefore might represent this population or those who have social challenges that contribute to withdrawing, such as divorce or medical leave. Fewer alumni responses than expected were received as well, with an indeterminate response rate, since alumni emails were the only available mode of contact and it is unlikely that all alumni regularly check these accounts. A solution to this would be surveying fourth-year students before they graduate to obtain their personal email addresses or phone numbers as their preferred modes of contact. Even with fewer alumni than students, it is probable that one alumnus can meet with multiple students.

There are several points for improving alumni recruitment as the network is further developed. At least at this institution, more alumni who identify as Hispanic/Latinx should be recruited to coordinate with student demand. Similarly, alumni can be recruited to address additional student concerns like military commitments or first-generation challenges. There was also a greater student demand for discussing neurodivergence, which can be addressed with recruitment and may partially be explained by an increase in the prevalence of adult diagnoses of attention-deficit/hyperactivity disorder [17].

The wording of the survey itself could be clarified as well. For example, there were more students than alumni who selected “becoming a parent,” but this item could be interpreted as wanting a family, already having a family, or having a child while in school.

Student mental health deserves special attention given that “depression/anxiety” was the most reported experience. First, depression and anxiety should be listed as separate experiences to clarify which one a student is self-reporting. Second, the high level of student interest begs the question of how to prepare the alumni for difficult conversations. While alumni are encouraged to share their own experiences, very few will have any training in psychotherapy and are not expected to take on the role of a therapist within this context. One solution could be to give alumni a list of therapy and psychiatric resources for students when they are accepted into the network. This way, alumni who identify acute mental health needs can offer these resources to students. At this stage, it is difficult to predict what other scenarios mentors might encounter. An annual feedback survey of network alumni might help identify if there are encounters for which alumni felt unprepared. Based on their responses, additional tools can be offered.

Taken together, this student-accepted idea addresses a need for preemptive support that is not currently offered at our institution. Next steps include the creation of an alumni database accessible on the student landing page. Students can filter the database by demographics or experience and contact alumni. The acceptability of this resource can be tracked with online usage and a follow-up survey after meetings occur. Due to cohort-specific characteristics, there is tremendous opportunity to explore this network as a resource at other medical programs to assess generalizability and support students through challenges.

## Data Availability

Data is available upon request from the corresponding author.
